# Acute Rheumatism and Valvular Disease

**Published:** 1900-03-24

**Authors:** 


### ACUTE RHEUMATISM AND VALVULAR DISEASE.

At the last meeting of the Clinical Society Dr. Richard Caton read a paper on the prevention of valvular disease in acute rheumatism which led to a somewhat interesting review by various speakers of the various methods in use in the treatment of the disease. Dr. Caton looked upon the prevention of injury to the heart as the first object to be aimed at. Rheumatism in itself was comparatively unimportant, and the case must be regarded first and before all as one of impending peril to the heart, endangering the whole life of the patient. The treatment which he recommended, after using it in nearly 500 cases of acute rheumatism, consisted in the following three measures: (1) Absolute rest in bed for several weeks; (2) the application of a series of blisters, in size somewhat less than a florin, one at a time, in the course of the first, second, third, and fourth dorsal nerve, a small poultice being applied after each blister; (3) the administration of sodium or potassium iodide in eight or ten grain doses thrice daily, with, in some cases, small doses of mercury. Dr. Caton believed that this treatment was only of use if commenced early—say within two or three weeks. In long-standing cases it was absolutely useless. Many speakers joined in the discussion. The one thing on which they were all agreed was the necessity for enforcing prolonged rest, all of which is true enough, albeit a little old.

